# Metabolic Changes in Minnan Pickled Radish During Long-Term Aging Revealed by Widely Targeted Metabolomics

**DOI:** 10.3390/foods15213942

**Published:** 2026-11-03

**Authors:** Jianhua Xie, Xuming Deng, Jinling Hong, Yuetao Chen, Yi Zheng, Zuhuan Li, Jie Pang

**Affiliations:** 1 College of Food Engineering, Zhangzhou Institute of Technology, Zhangzhou 363000, China; 2 Zhangzhou Food Industrial Technology Research Institute, Zhangzhou 363000, China; 3 School of Synthetic Biology and Biomanufacturing, Tianjin University, Tianjin 300072, China; 4 College of Food Science, Fujian Agriculture and Forestry University, Fuzhou 350002, China; pang3721941@163.com

**Keywords:** Minnan pickled radish, aging fermentation process, pathway mechanism analysis

## Abstract

Minnan pickled radish is a traditional aged fermented food with significant cultural and traditional value. In this study, we employed widely targeted metabolomics to characterize the dynamic metabolic profiles of Minnan pickled radish across five aging stages (0 to 8 years). Among the 339 core set, amino acids and their derivatives, alkaloids, and lipids were depleted at every aging stage relative to MPR0, whereas phenolic acids and organic acids responded divergently, with the upregulated subset accumulating progressively and peaking at MPR8. KEGG pathway analysis revealed pronounced carbon metabolic reprogramming at year 8: upregulated metabolites were primarily enriched in pyruvate metabolism and related pathways, whereas downregulated metabolites were associated with starch and sucrose degradation. Based on these findings, we proposed a metabolomics-driven model that establishes a conceptual framework linking carbohydrate catabolism to metabolic remodeling, providing a foundation for quality control of traditional fermented vegetables.

## 1. Introduction

Radish (*Raphanus sativus* L.), a cruciferous herb widely cultivated in China, is rich in nutrients such as dietary fiber, minerals, vitamins, proteins, phenolic acids, and anthocyanins [1]. Fermentation, a traditional method for food preservation, enhances food quality, flavor, and nutritional value [2]. Minnan pickled radish (MPR), known locally as “Laocai Pu” (aged radish) or “black radish” due to its dark appearance, is a traditional fermented food from the Minnan region of Fujian and the Chaoshan area of Guangdong, China [3]. Unlike the brine-based pickling commonly used in northern China, which relies on rapid lactic acid fermentation in a high-moisture environment, the Minnan process employs a distinctive solid-state fermentation involving repeated salting, sun-drying, pressing, and long-term aging in sealed earthen jars. This unique low-moisture, high-salt, multi-year aging approach fundamentally differentiates its microbial ecology and metabolic pathways from those of typical northern pickles, leading to a more complex and slowly evolving flavor profile. During curing and fermentation, radishes undergo complex biochemical reactions that alter their sensory properties, texture, and nutrient composition [4]. For instance, the levels of reducing sugars, organic acids, and free amino acids increase during the fermentation of dry-salted radishes [5]. Kumakura et al. [6] reported elevated γ-aminobutyric acid (GABA), polyunsaturated fatty acids, and branched-chain amino acids in fermented radishes, contributing to their unique flavor and enhanced nutritional profile. Aged MPR is traditionally consumed for its perceived health benefits, which increase with aging time [7]. These claims remain experimentally unverified. Moreover, the dynamic metabolite changes occurring during MPR fermentation have rarely been studied, necessitating a systematic investigation of its metabolic evolution.

Metabolomics, a cornerstone of systems biology alongside genomics, transcriptomics, and proteomics [8], enables comprehensive analysis of endogenous metabolites and identification of affected metabolic pathways [9]. Liu et al. [10] employed untargeted metabolomics to reveal the dynamic changes in metabolites and their association with sensory quality in pickled radishes across different aging years. However, this approach predominantly captures relatively abundant metabolites, potentially overlooking low-abundance yet functionally significant compounds. This limitation is particularly pertinent to MPR, as its unique solid-state fermentation and prolonged aging may produce low-concentration bioactive metabolites that remain unexplored. Widely targeted metabolomics, integrating the advantages of targeted and untargeted approaches, offers high sensitivity, broad coverage, and the capacity to identify thousands of metabolites, making it ideal for global nutrient profiling [11,12]. For example, Qi et al. [13] used this technique to reveal that anthocyanin downregulation and hydrolysis of gallocatechin and flavonol glycosides reduce astringency in Ampelopsis grossedentata, while geraniol fluctuations enhance aroma. Similarly, Dong et al. [14] demonstrated distinct accumulation patterns of lipids, amino acids, vitamins, phenolic acids, flavonoids, and glucosinolates across Chinese cabbage varieties, with phenolic acids as key differential metabolites. Despite growing applications of widely targeted metabolomics in food science, few studies have focused on dynamic metabolite variations and key pathways in radish fermentation. Notably, the aging of Chaoshan preserved radish (“Laocaifu”), a closely related aged radish product, has recently been reported to progressively accumulate acids, esters, and aging biomarkers [15], yet the underlying metabolic pathway dynamics remain largely unexplored.

Here, using UPLC-MS/MS-based widely targeted metabolomics, we systematically investigated metabolite distribution, accumulation differences, and potential transformation pathways during MPR aging. We further resolved the stepwise reorganization of central metabolic pathways—notably the sugar–organic acid–ester cascade that links sugar depletion to organic-acid accumulation and the formation of volatile-ester precursors—thereby providing a more systematic, pathway-level view of radish aging and a theoretical foundation for evaluating its nutritional value and comprehensive utilization.

## 2. Materials and Methods

### 2.1. Materials and Reagents

MPR samples were provided by Fujian Renchang Food Co., Ltd. (Zhangzhou, China). Reagents included methanol and acetonitrile (HPLC grade, Merck KGaA, Darmstadt, Germany), formic acid (HPLC grade, Shanghai Aladdin Biochemical Technology Co., Ltd., Shanghai, China), and ultrapure water prepared using a Direct-Pure adept UV ultrapure water system (RephiLe Bioscience Ltd., Shanghai, China).

### 2.2. Methods

#### 2.2.1. Sample Extraction

**Figure 1:**
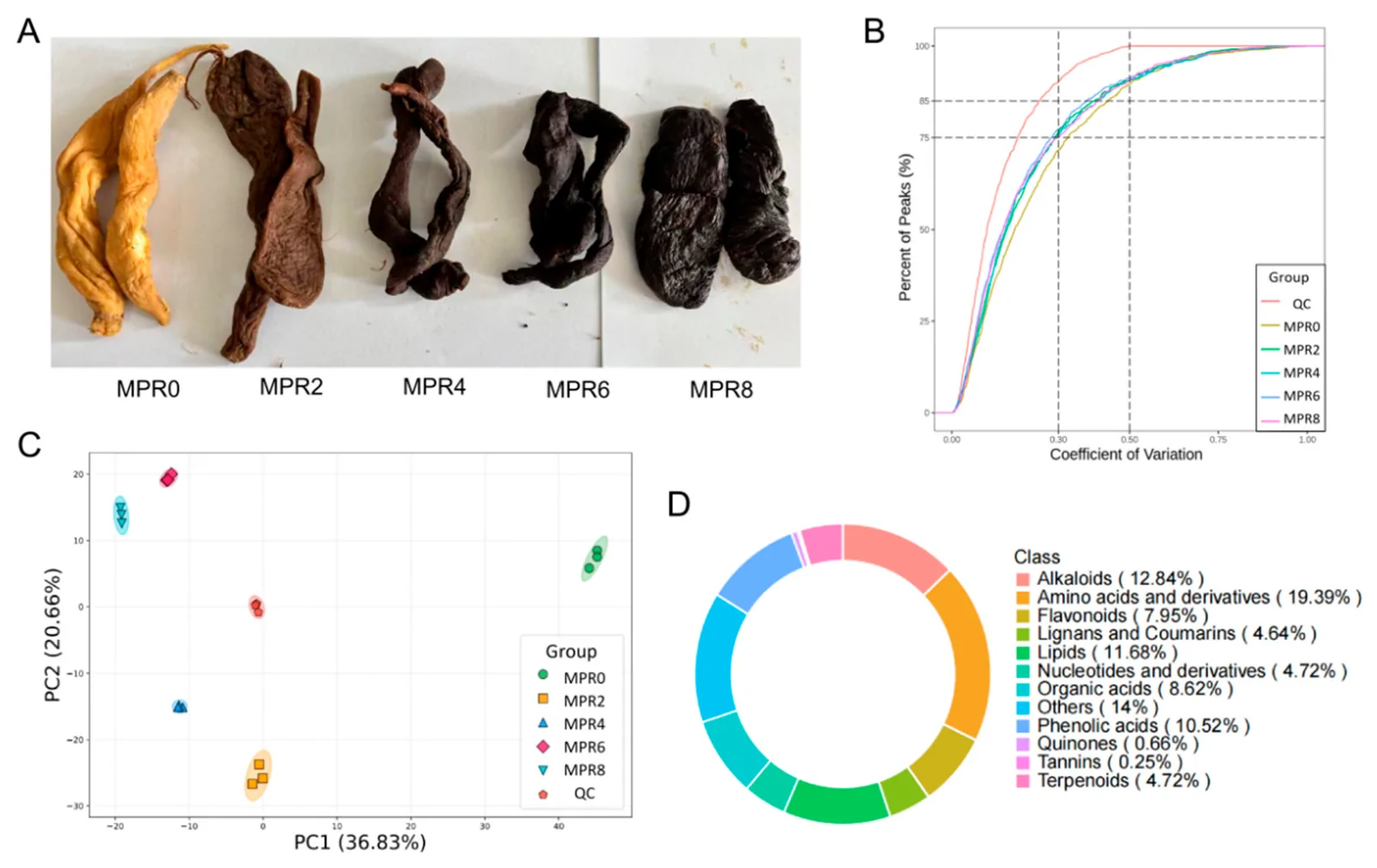
Quality control validation and metabolite profiling of Minnan pickled radish (MPR) during prolonged storage. (**A**) Physical appearance of MPR samples at different fermentation stages (MPR0, MPR2, MPR4, MPR6, and MPR8). (**B**) Empirical cumulative distribution function (ECDF) analysis of the coefficient of variation (CV) distribution for quality control (QC) and sample groups. (**C**) Principal component analysis (PCA) score plot showing distinct metabolic separation among fermentation stages. (**D**) Classification of the 1207 identified metabolites into 12 major chemical categories.

All samples of MPR were provided by Fujian Renchang Food Co., Ltd., China (Figure 1A). The radishes were produced locally from December to January of the following year (ambient temperature: 10–20 °C). The origin, variety, and quality of the raw materials (fresh white radishes, *Raphanus sativus* L.) were standardized, and processing was carried out according to established technical parameters in the same facility and workshop. The production process involved washing and sun-drying fresh radishes (to ~20% weight loss), rubbing with 5% sea salt and pressing in vats overnight, a second sun-drying (to ~40% weight loss), re-rubbing with an additional 6% sea salt and pressing, and repeated sun-drying by day and re-brining by night cycles (to ~70% weight loss); subsequently, the radishes were packed into sealed earthenware jars for one month and then aged to the finished product. Samples were collected after 0 (MPR0), 2 (MPR2), 4 (MPR4), 6 (MPR6), and 8 (MPR8) years of aging. MPR0 corresponds to the product at the time of sealing into the jars, immediately after the salting, sun-drying, pressing and one month of jar sealing described above (i.e., the onset of aging, aging time zero). For each aging stage, three independent earthenware jars from the same production year were sampled, giving n = 3 biological replicates per stage. All samples were immediately frozen in liquid nitrogen and lyophilized in a vacuum freeze-dryer, then ground into a fine powder (30 Hz, 1.5 min) and stored at −80 °C until further analysis. A 50 mg aliquot of lyophilized powder was mixed with 1200 μL of pre-chilled (−20 °C) 70% methanol aqueous solution containing the internal standard 2-chlorophenylalanine (J&K Scientific, Beijing, China, 98% purity, CAS 103616-89-3) at 1 ppm (mg/L), which served for retention-time alignment and to monitor instrument performance and was not used to derive absolute concentrations. The mixture was vortexed every 30 min (30 s per cycle, 6 cycles total), centrifuged at 12,000 rpm for 3 min, and filtered through a 0.22 μm membrane. The supernatant was stored in vials for UPLC-MS/MS analysis.

#### 2.2.2. Widely Targeted Metabolite Detection

Chromatographic separation was performed on an ExionLC™ AD ultra-performance liquid chromatography (UPLC) system coupled with a tandem mass spectrometry (MS/MS) system (SCIEX, https://sciex.com.cn/). Separation was achieved on an Agilent SB-C18 column (1.8 µm, 2.1 mm × 100 mm) maintained at 40 °C. The mobile phase consisted of ultrapure water containing 0.1% formic acid (solvent A) and acetonitrile containing 0.1% formic acid (solvent B), delivered at a flow rate of 0.35 mL/min. The gradient program was as follows: 5% B at 0 min, linearly increased to 95% B over 9 min and held for 1 min, then returned to 5% B at 11.1 min and re-equilibrated for 2.9 min. The injection volume was 2 µL. Mass spectrometric analysis was performed using an electrospray ionization (ESI) source operating at 550 °C with ion spray voltages of 5500 V (positive mode) and −4500 V (negative mode). Ion source gas I, gas II, and curtain gas were set at 50, 60, and 25 psi, respectively. Data acquisition was performed in multiple reaction monitoring (MRM) mode using a triple quadrupole mass analyzer. Declustering potential and collision energy for each MRM ion pair were individually optimized. Metabolite identification was based on an in-house database (MWDB, Metware Database) using MS/MS spectral matching, after removing isotope signals, adduct ions (K^+^, Na^+^, NH_4_^+^), and fragment ions derived from larger precursor molecules. Peak area integration and cross-sample alignment were performed for quantification, following established protocols [16]. No standard-curve calibration or absolute quantification was performed; accordingly, all abundance values are relative peak areas, and every abundance statement in this study refers to the change in a given metabolite across aging stages (log_2_FC versus MPR0) rather than to a cross-compound comparison of content.

Metabolite identification confidence was assigned according to the identification criteria of the widely targeted metabolomics platform: Level 1 (identified): the MS/MS spectrum (all fragment ions) and retention time (RT) matched the database entry with a matching score ≥ 0.7; Level 2 (putatively annotated): the MS/MS spectrum (all fragment ions) and RT matched the database entry with a matching score of 0.5–0.7; and Level 3 (putatively characterized): the Q1, Q3, RT, declustering potential (DP) and collision energy (CE) parameters were consistent with the database entry. Across Tables S1–S4, Level 3 annotations accounted for 37.5%, 53.3%, 50.8% and 33.3% of entries. Because MRM transitions cannot resolve stereoisomers or co-eluting isomers, D/L configurations could not be assigned, and metabolites sharing a transition (e.g., D-sucrose and D-trehalose, 341→119; D-mannose, D-glucose and D-galactose, 179→59) are reported as unresolved hexoses/disaccharides. Several annotations implausible for a radish product (e.g., N-ethylmaleimide, quinidine, 4-aminosalicylic acid and N-methyltetrahydropalmatine) are Level 3 or otherwise lack authentic-standard confirmation and may be false positives or contaminants; they were retained for completeness but are interpreted cautiously, and functional interpretation is limited to Level 1 compounds.

#### 2.2.3. Quality Control (QC) Samples

QC samples were prepared by pooling equal volumes of all extracts to assess methodological reproducibility. One QC sample was analyzed every 10 experimental samples to monitor instrumental stability throughout the sequencing run.

#### 2.2.4. Data Processing and Analysis

**Figure 2:**
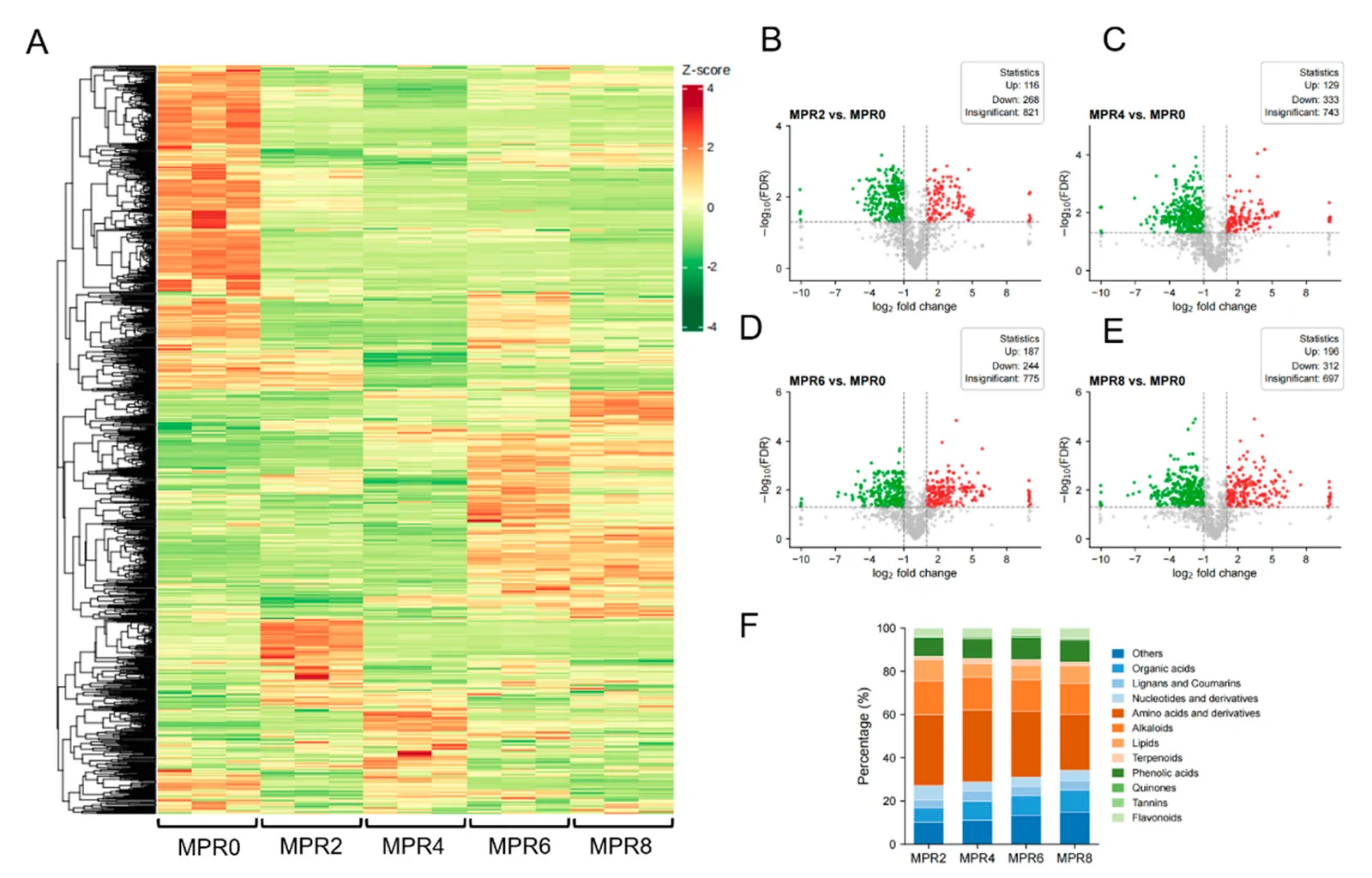
Biphasic metabolic trajectory and differential metabolite accumulation in Minnan pickled radish (MPR) during prolonged storage. (**A**) Hierarchical clustering heatmap of metabolite abundance across fermentation stages (MPR0, MPR2, MPR4, MPR6, and MPR8). (**B**–**E**) Volcano plots showing differentially abundant metabolites (DAMs) in MPR2 (**B**), MPR4 (**C**), MPR6 (**D**), and MPR8 (**E**) compared to MPR0. Red and green dots represent upregulated and downregulated metabolites, respectively, based on fold change and significance thresholds (grey dots: insignificant). (**F**) Stacked bar chart illustrating the proportional distribution of DAMs across major chemical categories.

Given the high-dimensional nature of metabolomics data, both univariate and multivariate statistical methods were employed to rigorously identify differentially abundant metabolites (DAMs). For each metabolite, an omnibus test of differences among the five aging stages was performed by one-way analysis of variance (ANOVA) on all 15 samples, and the resulting *p*-values were corrected for multiple testing using the Benjamini–Hochberg procedure (BH-FDR, n = 1200 metabolites). Fold change relative to MPR0 was calculated as FC = (group mean + 1)/(MPR0 mean + 1) and is reported as log_2_FC. Seven pairwise comparisons were performed using the same procedure: each aging stage versus MPR0 (MPR0 vs. MPR2, MPR0 vs. MPR4, MPR0 vs. MPR6, MPR0 vs. MPR8), together with the three additional consecutive-stage pairs (MPR2 vs. MPR4, MPR4 vs. MPR6, MPR6 vs. MPR8), all using Welch’s unequal-variance *t*-test (n = 3 per group) on untransformed peak areas, with Benjamini–Hochberg correction applied separately within each comparison. A metabolite was regarded as a DAM in a given comparison when it satisfied both |log_2_FC| ≥ 1 and the comparison-specific BH-FDR-adjusted *p* < 0.05; The consecutive-stage comparisons served only to establish whether the observed changes were progressive or phase-specific and to locate the critical stages. Metabolites meeting |log_2_FC| ≥ 1 in all four MPR0-referenced comparisons and showing a significant overall stage effect (five-stage ANOVA, BH-FDR < 0.05) were defined as the core set (n = 339). Throughout the manuscript, DAM counts and the volcano plots (Figure 2B–E and Figure S2) are based on the pairwise, comparison-specific BH-FDR values, whereas the FDR values in Tables S1–S4 derive from the five-stage ANOVA; the two address different null hypotheses and are therefore not interchangeable. Because a constant of 1 was added to both numerator and denominator, metabolites with zero intensity in one group give extreme ratios; log_2_FC values were therefore capped at ±10, and such cases are interpreted as presence/absence events rather than true fold changes (flagged in Tables S1–S4). No abundance normalization was applied, and analyses used raw peak areas. Because the product is substantially dehydrated during curing, all samples were lyophilized to constant dry weight before extraction, so that cross-stage comparisons reflect metabolite changes on a dry-matter basis and are not confounded by differences in water content. Unsupervised PCA was used instead to provide an overview of overall variation. PCA, hierarchical clustering and visualization tools (Venn diagrams, volcano plots) were performed using Metware Cloud (https://cloud.metware.cn/). Ring charts and bar plots were generated using Excel. Metabolite peak integration and correction were conducted using Analyst 1.6.3 software. Pathway annotation and enrichment analysis of DAMs were performed using the KEGG database. Enrichment significance was assessed by a hypergeometric test against the KEGG reference compound set (background, N = 5036 KEGG-annotated compounds) and visualized on the KEGG plant pathway map; only pathways containing at least one DAM were considered.

#### 2.2.5. 2,2-Diphenyl-1-picrylhydrazyl (DPPH) Radical Scavenging Activity Assay

The DPPH assay was performed as previously described [17]. First, 1 g of freeze-dried pickled radish sample was extracted with 70% methanol by ultrasonication at 60 °C for 60 min. After filtration, the extract was diluted to 50 mL. The same freeze-dried powder as used for metabolomics was employed; the DPPH extraction used 1 g of freeze-dried sample made up to 50 mL with 70% methanol (a sample-to-solvent ratio of 1:50, *w*/*v*, i.e., 20 mg dry matter per mL), whereas the metabolomics extraction used 50 mg in 1200 μL of the same solvent (≈41.7 mg dry matter per mL), so that both assays were performed on comparable dry-matter bases. A 1 mL aliquot was mixed with 2 mL of DPPH ethanol solution (0.1 mmol/L), incubated in the dark at room temperature for 30 min, and the absorbance was measured at 517 nm. Ascorbic acid (Vc, 0.01 mg/mL) served as the positive control. Because the extract (1 g in 50 mL) and the Vc solution (0.01 mg/mL) differ markedly in concentration, the DPPH results express relative antioxidant capacity among stages rather than an absolute potency. The scavenging rate (%) was calculated as Y = [1 − (A_1_ − A_2_)/A_3_] × 100, where A_1_, A_2_, and A_3_ represent the absorbances of the sample with DPPH, the sample without DPPH, and the blank, respectively. All measurements were performed in triplicate. Data are expressed as mean ± SD (n = 3). Differences among stages were assessed by one-way ANOVA followed by Tukey’s HSD post-hoc test, with *p* < 0.05 considered statistically significant.

## 3. Results and Discussion

### 3.1. QC Validation and Metabolite Profiling of Stored MPR

To investigate metabolite changes in MPR during prolonged storage, we employed widely targeted metabolomics. Over time, the radish color transitioned from yellowish-brown to black (Figure 1A). Technical reproducibility was first assessed by overlaying total ion chromatograms (TICs) of QC samples, which exhibited high overlap in retention times and peak intensities (Supplementary Figure S1), confirming stable instrument signals. Data reliability was further validated via ECDF analysis of CV distribution (Figure 1B): the QC group showed superior stability, with >85% of peaks having CV ≤ 0.5 and >75% having CV ≤ 0.3, while MPR0–MPR8 samples displayed comparable reproducibility, converging with the QC curve at higher CV thresholds. Principal component analysis (PCA, Figure 1C) revealed clear metabolic separation among all five stages: MPR0 clustered separately on the right (high PC1), whereas the four aging stages occupied distinct positions on the left, separated mainly along PC2—MPR2 at the bottom, MPR4 in the middle-lower region, and MPR6 and MPR8 in the upper region. QC samples aggregated near the center, confirming minimal technical variability and high data reliability.

Using the UPLC-MS/MS platform coupled with an in-house database, a total of 1207 metabolites were annotated and classified into 12 major chemical categories. Among the annotated metabolites, amino acids and derivatives were the most numerous class, accounting for 19.39%, followed by alkaloids (12.84%), lipids (11.68%), and phenolic acids (10.52%), with flavonoids (7.95%) and organic acids (8.62%) also contributing notably (Figure 1D). This comprehensive profile establishes a robust foundation for elucidating the chemical basis of pickled radish.

### 3.2. Biphasic Metabolic Trajectory and Differential Metabolite Accumulation in Mpr

As illustrated in the clustering heatmap (Figure 2A), the metabolic composition of pickled radish exhibited a biphasic trajectory over time. A sharp decline in metabolite abundance was observed in MPR2 and MPR4, which clustered distinctly away from the MPR0 baseline, reflecting intense biochemical degradation during the mid-fermentation stage. Notably, this downward trend reversed partially in the long-term samples: MPR6 and MPR8 formed a tight cluster separate from the MPR4 trough, and the stage-wise average abundance of the metabolites recovered from its minimum at MPR4.

Differential metabolite analysis (Figure 2B–E) corroborated this pattern. Compared to MPR0, the early fermentation stages (MPR2 and MPR4) were dominated by metabolite downregulation: 116 upregulated and 268 downregulated metabolites were detected in MPR2 (Figure 2B), and 129 upregulated and 333 downregulated in MPR4 (Figure 2C), indicating a sustained decline in metabolite levels. However, the later stages exhibited a rebound in metabolic activity: MPR6 versus MPR0 yielded 187 upregulated and 244 downregulated metabolites (Figure 2D), and MPR8 reached 196 upregulated and 312 downregulated metabolites (Figure 2E), underscoring notable biochemical restructuring during prolonged storage.

To establish whether these changes were progressive or phase-specific, each pair of consecutive stages was compared using the same criteria (Figure S2). MPR0→MPR2 (384 DAMs; 268 down/116 up) and MPR2→MPR4 (330; 192 down/138 up) were both down-dominated, whereas MPR4→MPR6 (353; 284 up/69 down) was the only upregulation-dominated transition, and MPR6→MPR8 (111; 72 down/39 up) was again down-dominated with markedly attenuated amplitude. These step-wise comparisons therefore locate the metabolic turning point at MPR6, where the depletion phase ends and the most extensive upregulated remodelling occurs, and identify MPR8 as the endpoint state with the greatest divergence from the fresh baseline (508 DAMs; Figure 2E). Years 6 and 8 are thus the critical stages of MPR aging.

Classification analysis (Figure 2F) further revealed that the differential metabolites were predominantly enriched in amino acids and their derivatives, alkaloids, phenolic acids, organic acids, and lipids, which together accounted for 69.5–74.7% of DAMs across the four aging stages. This distribution highlights that fermentation primarily drives the degradation of primary metabolites (e.g., amino acids, declining from 32.6% at MPR2 to 25.6% at MPR8), alongside the dynamic turnover of secondary metabolites (e.g., alkaloids, 15.6–14.4%, and phenolic compounds, 9.6–11.0%).

### 3.3. Core Differentially Abundant Metabolites and Their Dynamic Changes Across Major Chemical Classes in Mpr

**Figure 3:**
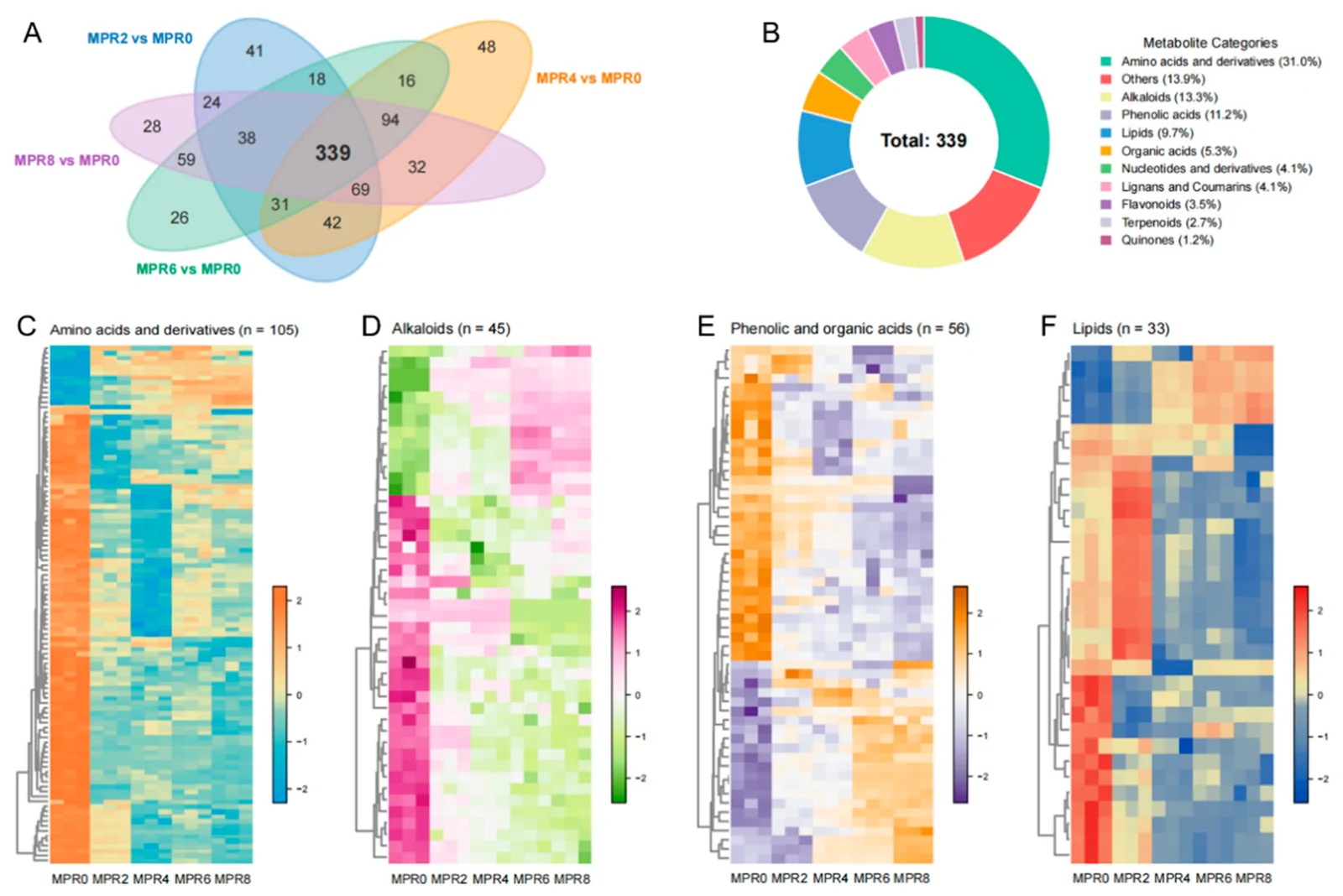
Dynamic changes and classification of core differential metabolites during the prolonged storage of Minnan pickled radish (MPR). (**A**) Venn diagram illustrating the overlapping and unique core sets across the four post-fermentation stages (MPR2, MPR2, MPR6, and MPR8) compared to MPR0. (**B**) Donut chart showing the proportional distribution of the 339 core set across major chemical categories. (**C**–**F**) Heatmaps depicting the dynamic abundance patterns of the shared core set in amino acids and their derivatives (**C**), alkaloids (**D**), phenolic acids and organic acids (**E**), and lipids (**F**).

To clarify the association between fermentation duration and metabolite abundance, we defined a core set as those with |log_2_FC| ≥ 1 in all four comparisons against MPR0 (MPR2, MPR4, MPR6 and MPR8 vs. MPR0) and a significant overall stage effect (five-stage ANOVA, BH-FDR < 0.05), yielding 339 metabolites (Figure 3A). These metabolites were classified mainly into five categories—amino acids and their derivatives, alkaloids, phenolic acids, organic acids, and lipids—which together accounted for 70.5% of the core set (Figure 3B).

Amino acids and their derivatives constituted the largest group of differential metabolites, playing critical roles in flavor development and bioactive properties [18,19]. Their abundance was consistently lower than in MPR0 at every aging stage, with 88.6% (93/105) of shared amino acids and derivatives exhibiting significantly lower levels post-fermentation, while only 12 were upregulated (Figure 3C). This reduction likely results from Maillard reactions and hydrolysis that convert free amino acids into biogenic amines and volatile flavor compounds during solid-state fermentation [10,20]. For example, several amino acids associated with sweetness, astringency, and bitterness [21,22]—including L-tryptophan (log_2_FC: −4.11, −4.67, −4.69, and −4.39 for MPR2, 4, 6, and 8 vs. MPR0, respectively), L-arginine (log_2_FC: −1.15, −3.01, −2.18, and −1.79), L-lysine (log_2_FC: −2.17, −2.15, −2.36, and −2.12), and L-histidine (log_2_FC: −1.67, −2.79, −2.94, and −2.32)—were markedly reduced post-fermentation (Table S1), potentially explaining the attenuation of these taste attributes [10]. Conversely, a distinct subset of metabolites displayed a resurgence in later stages. Notably, L-tyrosine (log_2_FC: −2.32, −1.64, −1.50, and −1.15), a precursor to clove-like aroma compounds [23], decreased in MPR2 but rebounded in MPR6 and MPR8 (Table S1), suggesting its potential involvement in flavor maturation. Similarly, cyclic peptides such as cyclo(valine-alanine) (log_2_FC: +1.73, +2.65, +2.92, and +3.02) accumulated progressively during fermentation. Known for antimicrobial, antimalarial, and anticancer properties [19], these peptides may contribute to both the flavor and bioactivity of aged MPR. Furthermore, a series of dipeptides and tripeptides—including L-leucyl-L-leucine (log_2_FC: −1.84, −1.80, −1.72, and −2.22) and L-valyl-L-leucine (log_2_FC: −1.88, −1.76, −1.40, and −2.03)—exhibited relatively stable abundance in MPR6 and MPR8 compared to earlier timepoints (Table S1). These small peptides are reported to possess antioxidant and antihypertensive activities and may serve as markers of protein hydrolysis during extended fermentation.

Of the 45 alkaloids in the core set, 66.7% (30/45) were consistently downregulated while 28.9% (13/45) were upregulated across all four post-fermentation timepoints; the remaining two changed direction between stages (Figure 3D). As detailed in Table S2, seven of eight indole alkaloids (Plumerane subclass) were markedly reduced. Indole-5-carboxylic acid (log_2_FC: −2.74, −4.67, −4.33, and −5.05) and 3-indoleacrylic acid (log_2_FC: −4.31, −4.27, −6.11, and −5.24)—both natural antioxidants capable of scavenging free radicals and inhibiting polyphenol oxidase (PPO) activity—were significantly depleted. Their sustained decline likely accelerates browning and color darkening during prolonged storage. Indole-3-cyano-6-O-glucoside (log_2_FC: −2.34, −3.62, −3.91, and −4.60), a potential precursor of bitter cyanogenic compounds, also decreased progressively, which may mitigate bitterness development. Conversely, indole-3-carboxaldehyde (log_2_FC: −1.45, −1.82, −2.41, and −2.80), a volatile that emits jasmine-like floral notes at low concentrations but pungent off-odors at elevated levels, exhibited a moderate yet consistent decline, potentially contributing to a refined flavor profile. Phenolic amides, including feruloylcholine glucoside (log_2_FC: −3.89, −3.70, −4.45, and −5.17)—a compound implicated in cell wall integrity through polysaccharide cross-linking—were similarly reduced, likely contributing to textural softening and loss of crispness.

Phenolic acids and organic acids, both classified under the broader category of organic acids, play critical roles in shaping flavor profiles, regulating pH, and conferring bioactive properties such as antioxidant, antimicrobial, anticancer, and hypoglycemic effects [24,25,26,27]. A total of 56 organic acids (38 phenolic acids and 18 organic acids) in the core set were identified post-fermentation. Among these, 31.6% (12/38) of phenolic acids were upregulated and 60.5% (23/38) downregulated (Figure 3E), while among the remaining organic acids, 9 were downregulated and 6 upregulated. Within the upregulated subset, key upregulated phenolic acids increased progressively, peaking in MPR8: gentisic acid (log_2_FC = 3.56, 3.65, 5.50, and 5.67), protocatechuic acid (log_2_FC = 3.85, 3.74, 5.38, and 5.84), 4-aminosalicylic acid (log_2_FC = 3.87, 3.14, 6.01, and 6.06) and salicylic acid (log_2_FC = 3.62, 2.98, 4.97, and 5.10) (Table S3). Similarly, several organic acids accumulated with prolonged fermentation, with their upregulation peaking at MPR6 or MPR8 (Table S3), which may partly reflect ongoing microbial metabolism, although non-enzymatic pathways cannot be excluded [28]: 3-hydroxypropionic acid (log_2_FC = 1.30, 1.81, 2.31, and 2.54), 2-hydroxyisobutyric acid (log_2_FC = 1.18, 1.65, 2.29, and 2.45), 5-hydroxymethyl-2-furancarboxylic acid (log_2_FC = 3.06, 4.35, 5.82, and 5.32), tropic acid (log_2_FC = –1.04, 2.96, 2.95, and 5.18), and acetoxyacetic acid (log_2_FC = 3.29, 3.06, 5.01, and 4.13). Functionally, salicylic acid has been reported to alleviate intestinal inflammation by inhibiting inflammatory factors (e.g., COX-2) [29], while protocatechuic acid exhibits antimicrobial activity in vitro, extending shelf life [30]. Gentisic and salicylic acids scavenge superoxide anions (O_2_^−^) and hydroxyl radicals (·OH), and are reported to contribute to health benefits such as anti-aging and anticancer effects [31]. Short-chain hydroxy acids like 3-hydroxypropionic acid enhance sourness, while tropic acid and 5-hydroxymethyl-2-furancarboxylic acid contribute to volatile flavor compounds and non-enzymatic browning [32,33]. Collectively, these dynamic shifts suggest potential nutritional and sensory relevance of fermented radish.

Lipid metabolism plays a pivotal role in determining product quality. Differential lipids encompassed 20 free fatty acids (FFAs), 12 lysophospholipids (7 lysophosphatidylethanolamines and 5 lysophosphatidylcholines) and 1 sphingolipid, totaling 33 species in the core set (Figure 3F, Table S4). Notably, all lysophospholipids—including LysoPE 18:2 (log_2_FC = 5.03, −1.85, −2.76, and −2.68), LysoPC 16:0 (log_2_FC = 2.37, −1.56, −2.06, and −4.69), and LysoPE 16:0 (log_2_FC = 2.44, −2.62, −3.36, and −5.04)—were markedly elevated at MPR2 yet subsequently declined, suggesting a transient lipolytic burst early in fermentation. Concurrently, five free fatty acids were upregulated at all four timepoints; most reached their highest relative levels at MPR6, whereas 12,13-dihydroxyoctadec-9-enoic acid and hexadecanedioic acid peaked at MPR8 (Table S4): 12,13-dihydroxyoctadec-9-enoic acid (log_2_FC = 1.27, 3.82, 4.41 and 5.00), 9-hydroperoxy-9Z,11E-octadecadienoic acid (log_2_FC = 2.53, 4.72, 5.75 and 5.42), 7S,8S-dihydroxyoctadeca-9,12-dienoic acid (log_2_FC = 3.22, 5.51, 6.49 and 6.10), hexadecanedioic acid (log_2_FC = 1.62, 3.71, 4.86 and 5.51) and 14,15-DiHETE (log_2_FC = 3.16, 1.08, 3.98, and 4.40). Lipid hydrolysis during fermentation generates flavor precursors—such as volatile aldehydes and ketones derived from polyunsaturated fatty acid oxidation—which may contribute to the aroma profile of aged radish [24].

### 3.4. Antioxidant Capacity of Aged MPR and Its Association with Phenylalanine Metabolism

**Figure 4:**
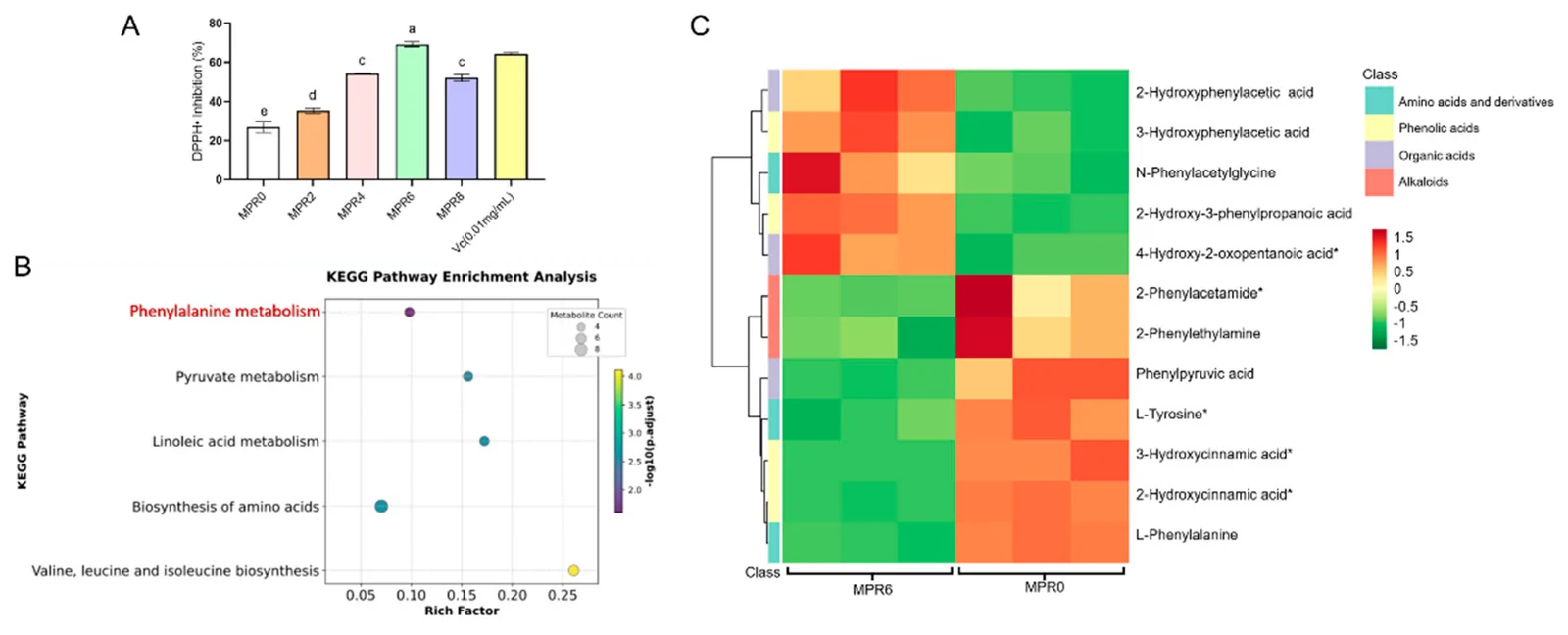
Antioxidant capacity of aged Minnan pickled radish (MPR) and its association with phenylalanine metabolism. (**A**) DPPH radical scavenging activity of MPR at different fermentation stages, with vitamin C (Vc) as a reference (not included in the statistical comparison). Data are mean ± SD (n = 3); different lowercase letters indicate significant differences (one-way ANOVA, Tukey’s HSD, *p* < 0.01). (**B**) KEGG pathway enrichment analysis of upregulated differentially abundant metabolites (DAMs) in MPR6 versus MPR0. (**C**) Heatmap showing the relative abundance of key metabolites related to phenylalanine metabolism in MPR6 versus MPR0.

To validate the long-term antioxidant efficacy of aged MPR, DPPH radical scavenging assays were conducted across different fermentation years (Figure 4A). The results showed that antioxidant capacity increased up to MPR6, which exhibited the strongest DPPH inhibition and was significantly higher than MPR0, MPR2 and MPR4 (*p* < 0.01), while MPR8 declined to a level comparable to MPR4.

Given the superior antioxidant activity observed in the MPR6 sample, we further investigated the underlying metabolic mechanisms by performing KEGG pathway enrichment analysis on the upregulated DAMs in MPR6 versus MPR0 (Table S5). Among the enriched pathways, phenylalanine metabolism was identified (Figure 4B). Prior studies have established that the activation of phenylalanine metabolism substantially enhances cellular antioxidant capacity through the accumulation of phenolic compounds and their derivatives, which are downstream products of the phenylpropanoid pathway [34]. To delineate the specific metabolic perturbations within this pathway, we mapped the expression patterns of related metabolites as a heatmap (Figure 4C). The MPR6 group showed marked upregulation of 2-hydroxyphenylacetic acid, 3-hydroxyphenylacetic acid, N-phenylacetylglycine, and 2-hydroxy-3-phenylpropanoic acid relative to MPR0. These compounds are catabolites or conjugates within phenylalanine metabolism and are generally weak radical scavengers, so they may contribute only modestly to the observed antioxidant activity. Given the progressive blackening of the product, Maillard-derived melanoidins—well-known antioxidants in aged, dark fermented products—are also likely to make a substantial contribution and warrant consideration alongside phenylalanine-derived phenolics [35]. Similarly, a recent study on kiwi wine showed that metabolite composition is closely linked to antioxidant properties and overall product quality [36].

### 3.5. Divergent Regulation of Central Carbon Metabolism in MPR8

**Figure 5:**
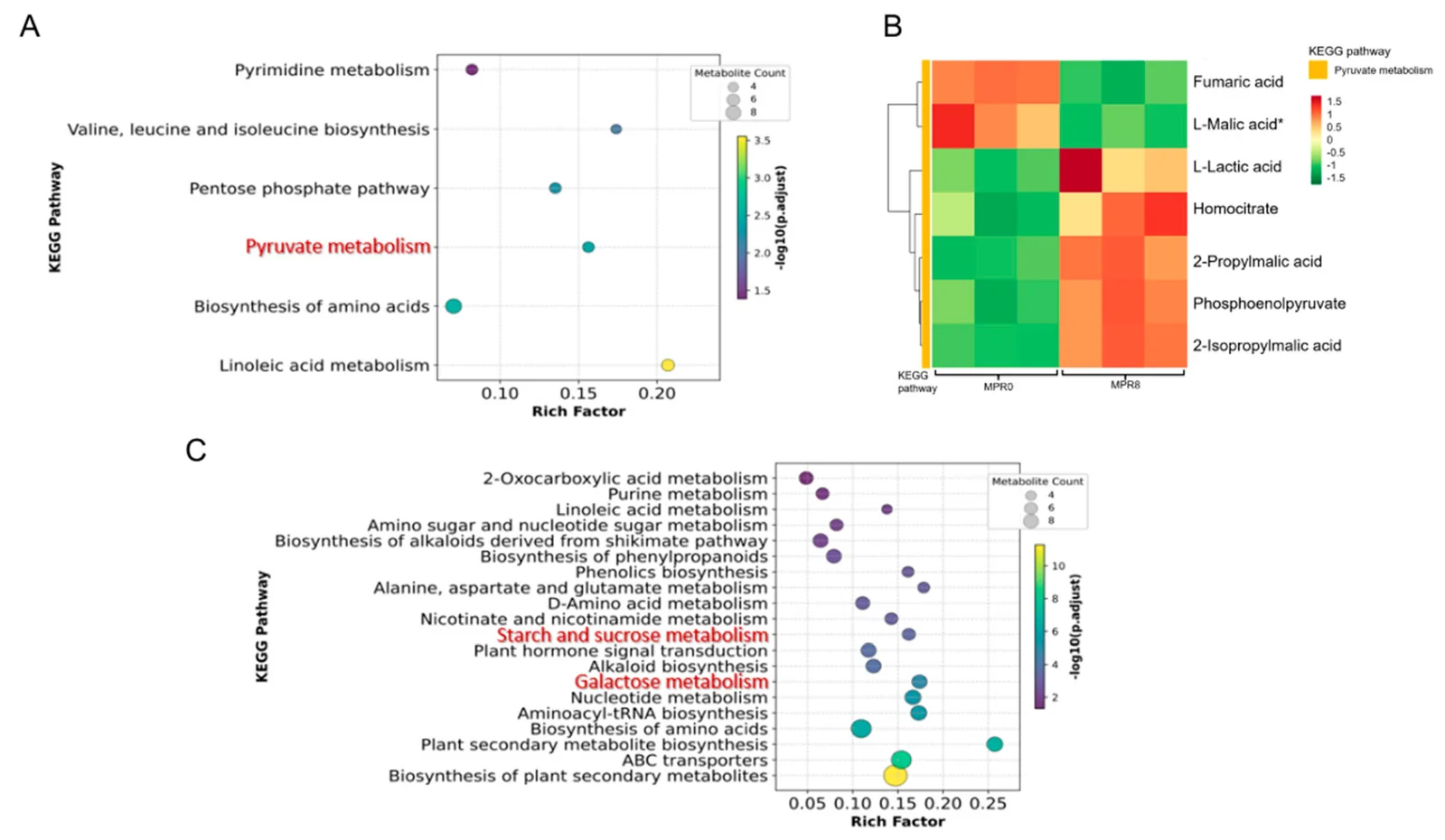
Divergent regulation of central carbon metabolism in 8-year-aged Minnan pickled radish (MPR8). (**A**) KEGG pathway enrichment analysis of upregulated differentially abundant metabolites (DAMs) in MPR8 compared to MPR0. (**B**) Heatmap showing the relative abundance of key metabolites in the pyruvate metabolism pathway between MPR8 and MPR0. (**C**) KEGG pathway enrichment analysis of the top 20 enriched pathways among the downregulated DAMs in MPR8 compared to MPR0.

To elucidate the metabolic remodeling underlying the unique quality of 8-year-aged MPR, a comparative analysis against the 0-year baseline was conducted. KEGG pathway enrichment analysis revealed distinct regulatory trends among the DAMs (Figure 5A,C).

Specifically, the upregulated metabolites in the MPR8 group were significantly enriched in several core carbon metabolic pathways (Figure 5A, Table S6). Among these, the pyruvate metabolism pathway attracted particular attention because of its relevance to flavor and nutritional quality [37]. Specifically, pyruvate links glycolysis to the formation of lactic acid, acetic acid, alcohol and esters, which directly contribute to the characteristic sour taste and overall sensory complexity of fermented radish [38,39]. Although pyruvate itself was not detected—most likely owing to the poor retention and ionization of this low-molecular-weight α-keto acid under the reversed-phase C18/negative-ion MRM conditions used—the marked accumulation of its immediate precursors and products, phosphoenolpyruvate and L-lactic acid (Figure 5B), is consistent with active pyruvate metabolism at this pathway. The accumulation of these intermediates is statistically associated with the formation of volatile esters, may also be related to energy production, and provides precursors for flavor- and bioactivity-related compounds [40,41]. Collectively, these findings indicate a coordinated remodeling of primary metabolism associated with redox balance and the synthesis of flavor- and nutrition-related compounds, in which pyruvate metabolism is likely to play a central role.

Conversely, the downregulated metabolites were significantly enriched in carbohydrate catabolic pathways, particularly starch and sucrose metabolism and galactose metabolism. (Figure 5C, Table S7). Carbohydrate catabolism constitutes a pivotal determinant of the sensory and nutritional evolution of aged pickled radish, as soluble sugars, organic acids, and derived metabolites collectively shape the taste profile during prolonged pickling [10,38]. Although fresh radish root contains relatively little starch, any starch present, together with storage carbohydrates, could be progressively hydrolyzed into maltose and glucose by endogenous and microbial amylolytic enzymes (α-amylase, β-amylase, and glucoamylase), potentially during the early fermentation phase [40]. However, in the absence of microbial community, viable-count or enzyme-activity data, this remains a hypothesis, and non-enzymatic hydrolysis cannot be excluded. The resulting hexoses, or the disaccharides themselves, may have been metabolized by lactic acid bacteria (or other halotolerant microbes) via glycolysis to generate pyruvate—the central precursor for lactic acid, acetic acid, ethanol, and ester formation [38,41]. Sucrose, a major disaccharide contributing to the sweet taste profile, is cleaved into glucose and fructose by invertase (sucrose-hydrolyzing enzymes), which are subsequently channeled into glycolysis [42,43].

### 3.6. Metabolic Progression Shaping Flavor and Nutritional Qualities in Aged Mpr

**Figure 6:**
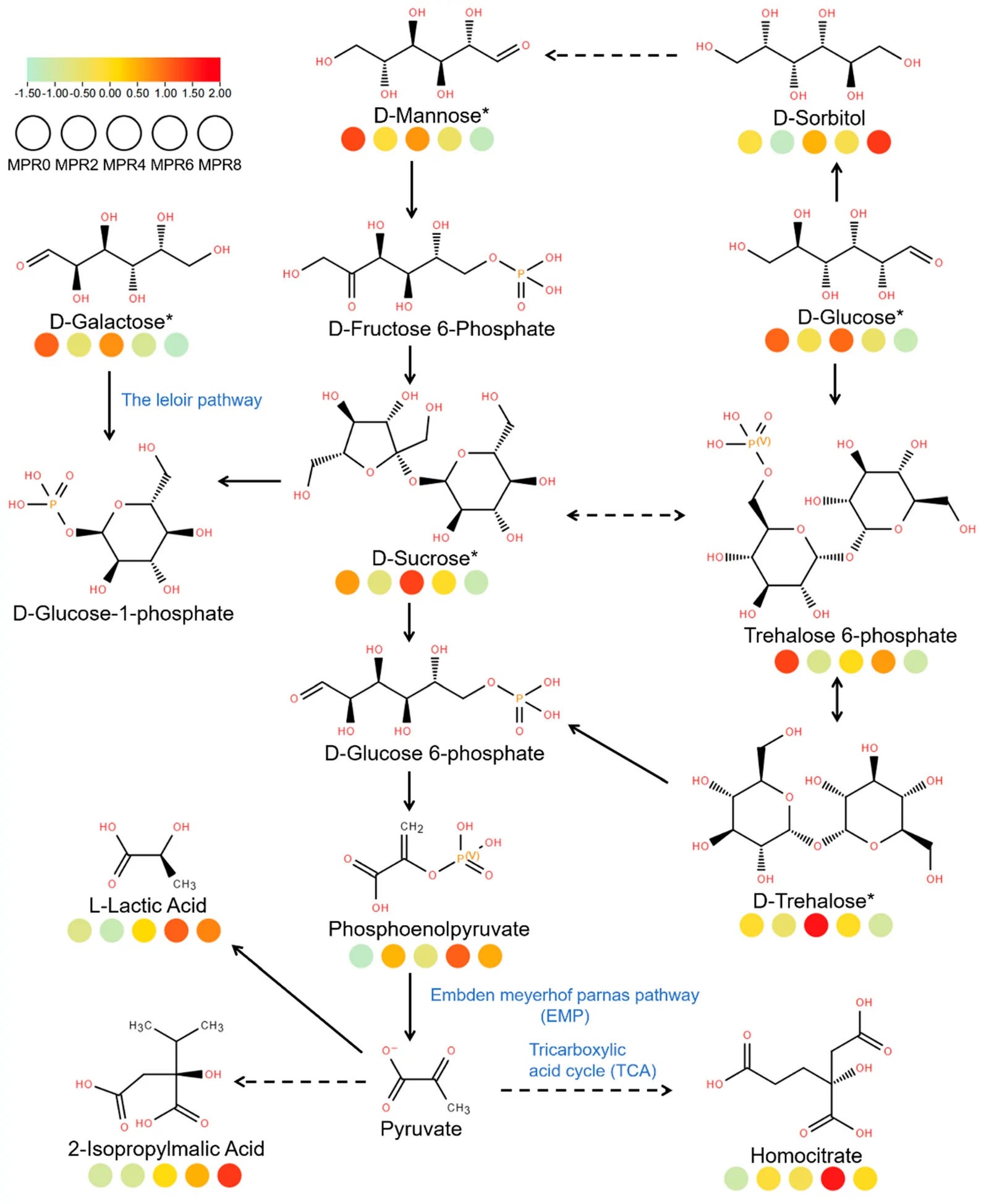
Proposed metabolic reactions determining flavor and nutritional quality in Minnan aged pickled radish (MPR). This schematic diagram illustrates the dynamic, stage-specific metabolic cascade that drives flavor formation across the five fermentation stages (MPR0–MPR8), where colored circles indicate the relative metabolite abundance. Asterisks mark metabolites that share a single MRM transition and are therefore reported as unresolved hexoses/disaccharides (D-sucrose/D-trehalose, 341→119; D-mannose/D-glucose/D-galactose, 179→59); most other intermediates shown are Level 2–3 annotations with unassigned D/L configurations, so this model is tentative and hypothesis-generating. The underlying peak-area values (mean ± SD) are provided in Table S8. Solid arrows indicate direct single-step enzymatic reactions; dashed arrows indicate indirect, multi-step, or inferred relationships based on KEGG pathway annotations.

Combined with the enrichment analysis results of key metabolic pathways in Figure 5A,C, we further constructed the pathway mechanism during the fermentation process of MPR. Figure 6 and Table S8 depict the dynamic and stage-specific metabolic progression driving flavor formation in aged MPR, illustrating the progressive redirection of carbon flux from primary storage carbohydrates toward characteristic flavor-active substances. Metabolomic profiling across the five fermentation stages (MPR0–MPR8) revealed distinct stage-specific abundance patterns of key intermediates, suggesting the progressive engagement of core metabolic pathways.

Under hyperosmotic and mildly acidic conditions, the mobilization of storage carbohydrates and the initiation of primary metabolism were first triggered: D-sucrose and D-trehalose decreased to 0.36-fold and 0.68-fold at MPR2 versus MPR0 and then transiently rebounded to their stage-wise maxima at MPR4 before declining again, while D-galactose levels concurrently declined to 0.58-fold, indicating that storage sugars were converted via hydrolysis or phosphorylation into key metabolic hubs, including D-glucose-6-phosphate, D-glucose-1-phosphate, and D-fructose-6-phosphate, with D-galactose further entering the Leloir pathway to replenish the cellular carbon pool. Subsequently, glycolytic flux was markedly enhanced, as evidenced by the substantial increase in phosphoenolpyruvate (5.03-fold from MPR0 to MPR2), suggesting accelerated carbon flow through the Embden-Meyerhof-Parnas pathway toward pyruvate accumulation and the expansion of the pyruvate pool. As a central node for the biosynthesis of organic acids and flavor compounds, the partitioning of carbon at the pyruvate node profoundly shaped the composition of end products: L-lactic acid increased to a peak at MPR6 (6.35-fold from MPR0) before declining to 4.77-fold at MPR8; similarly, homocitrate peaked at MPR6 before sharply declining to 0.29-fold at MPR8 relative to MPR6, while 2-isopropylmalic acid increased steadily (11.9-fold from MPR0 to MPR8). Biochemically, homocitrate and 2-isopropylmalic acid are formed from 2-oxoglutarate and 2-oxoisovalerate, respectively, via acetyl-CoA-dependent reactions rather than directly from pyruvate; their accumulation therefore reflects carbon flux downstream of the pyruvate node rather than a direct pyruvate-derived route. The accumulation of these organic acids may synergistically lower the system pH and enrich the sensory profile of the final product. Notably, 2-isopropylmalic acid, a branch-point metabolite in the leucine biosynthesis pathway, has been implicated in the formation of isoamyl alcohol and 3-methylbutyl esters, which are key aroma compounds previously reported in wine [44].

Overall, the flavor formation of aged MPR follows a continuous biochemical evolution. The marked decline of most sugars by the later stages, despite their transient rebound at MPR4, not only attenuates residual sweetness but also drives the accumulation of key organic acids that define the characteristic sour taste and serve as potential precursors for volatile esters responsible for fruity and floral aromatic notes [37]. The apparent sequential engagement of starch and sucrose metabolism, galactose metabolism, and glycolysis/pyruvate metabolism, together with the quantitative dynamics of these organic acids, suggests a mechanistic framework linking carbohydrate catabolism to characteristic flavor generation, a hypothesis that merits direct microbial and enzymatic validation.

## 4. Conclusions

This study employed widely targeted metabolomics to systematically characterize the dynamic metabolic landscape of MPR during 0 to 8 years of storage. The metabolic changes exhibited a biphasic trajectory: an initial decline in metabolite abundance during the early stage (MPR0–MPR4), followed by a partial rebound during the later stages (MPR6–MPR8). Among the 339 core set, amino acids and their derivatives, alkaloids, and lipids were depleted at every stage relative to MPR0, whereas phenolic acids and organic acids accumulated progressively and peaked at MPR8, consistent with a carbohydrate-driven reprogramming of central carbon metabolism toward the generation of flavor- and nutrition-related compounds. DPPH radical scavenging assays confirmed that antioxidant activity increased in a time-dependent manner, peaking at the MPR6 stage. These findings indicate that prolonged aging reshapes the metabolite profile in a stage-specific manner. Future studies integrating enzyme activity assays and targeted metabolic flux analysis are warranted to further validate the underlying regulatory mechanisms. This work provides a metabolic reference for quality control and aging-time-dependent optimization of traditional fermented vegetable products.

## Data Availability

All of the data included in this study are available upon request by contacting the corresponding author.

## Supplementary Materials

The following supporting information can be downloaded at: https://www.mdpi.com/article/10.3390/foods15213942/s1. Figure S1: Technical reproducibility assessment for the metabolomics analysis of MPR; Figure S2: Volcano plots showing differentially abundant metabolites (DAMs) in the four consecutive-stage comparisons: MPR0 vs. MPR2 (A), MPR2 vs. MPR4 (B), MPR4 vs. MPR6 (C), and MPR6 vs. MPR8 (D); Table S1: Differential abundances of amino acids and their derivatives in MPR across fermentation stages (0–8 years); Table S2: Differential abundances of alkaloids in MPR across fermentation stages (0–8 years); Table S3: Differential abundances of phenolic acids and organic acids in MPR across fermentation stages (0–8 years); Table S4: Differential abundances of lipids in MPR across fermentation stages (0–8 years); Table S5: KEGG pathway enrichment analysis of upregulated differentially abundant metabolites (DAMs) in MPR6 compared to MPR0; Table S6: KEGG pathway enrichment analysis of upregulated differentially abundant metabolites (DAMs) in MPR8 compared to MPR0; Table S7: KEGG pathway enrichment analysis of downregulated differentially abundant metabolites (DAMs) in MPR8 compared to MPR0; Table S8: Differential abundance of key metabolites underlying flavor and nutritional quality across five fermentation stages (0–8 years).
