# Supplementary material for: Metabolic Changes in Minnan Pickled Radish During Long-Term Aging Revealed by Widely Targeted Metabolomics
Source: Foods. 2026 Nov 3;15(21):3942. doi: 10.3390/foods15213942 (PMC13763023; doi:10.3390/foods15213942)
Supplement: Supplementary file 1 [file foods-15-03942-s001.zip › Supplementary Figure.pdf]

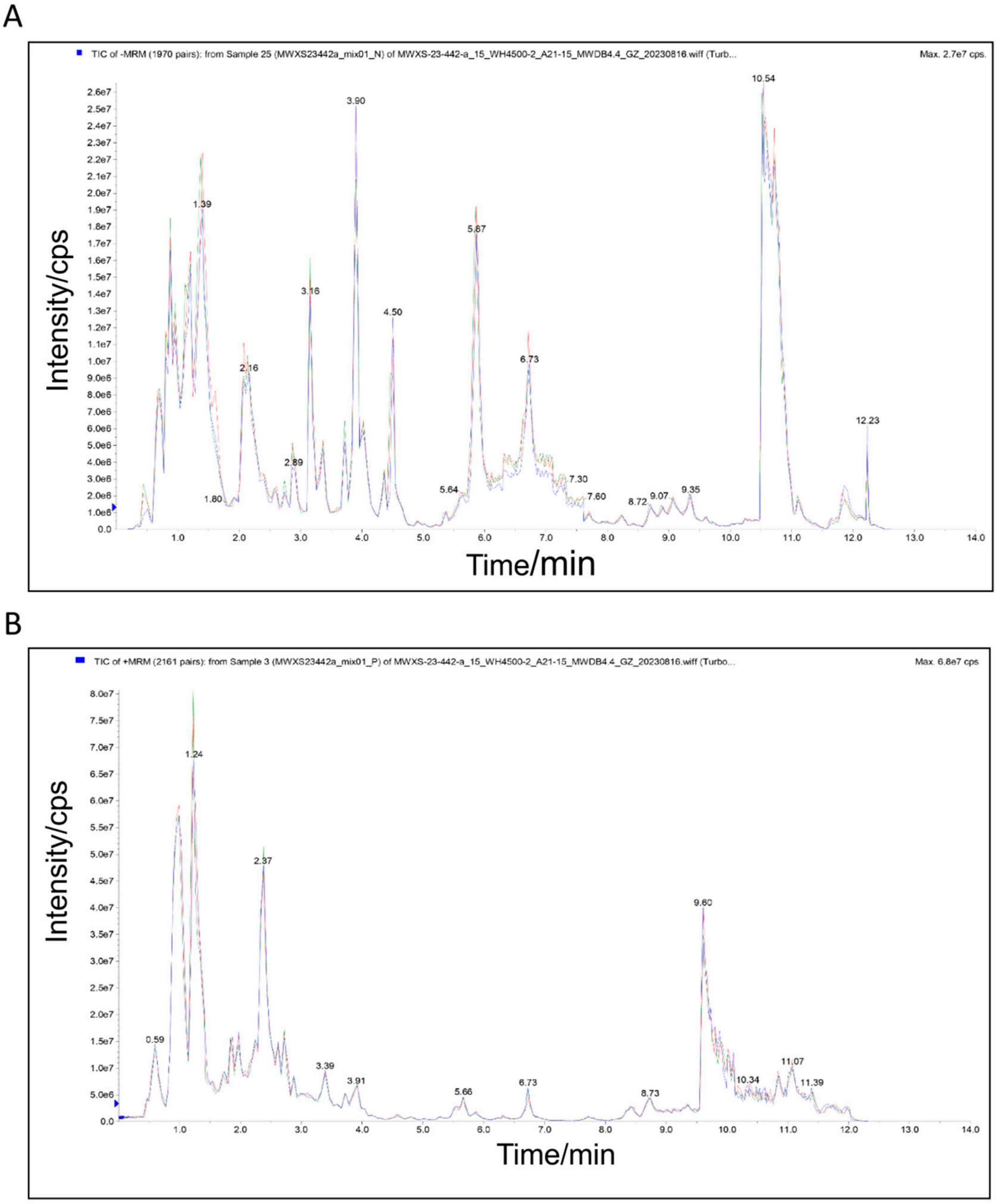


**Figure S1.** Technical reproducibility assessment of the metabolomics analysis. Overlay of total ion chromatograms (TICs) from quality control (QC) samples in (A) negative ion mode (-MRM, 1970 pairs) and (B) positive ion mode (+MRM, 2161 pairs).

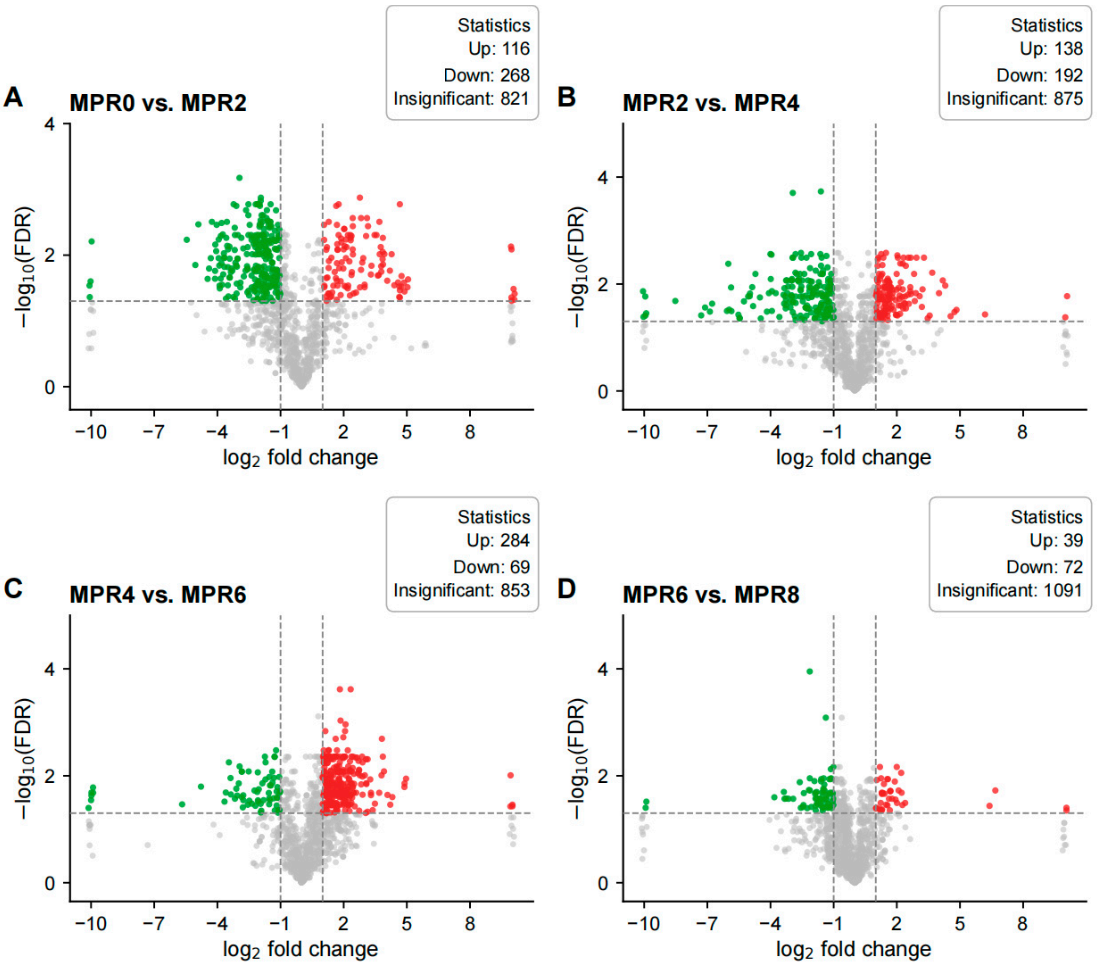


**Figure S2.** Volcano plots showing differentially abundant metabolites (DAMs) in the four consecutive-stage comparisons: MPR0 vs. MPR2 (A), MPR2 vs. MPR4 (B), MPR4 vs. MPR6 (C), and MPR6 vs. MPR8 (D). Each point represents one metabolite (n = 1,205–1,206). Red and green indicate metabolites meeting $|\log_2 FC| \geq 1$ and BH-FDR-adjusted $p < 0.05$ (Welch's unequal-variance t-test; Benjamini–Hochberg correction applied separately within each comparison); grey indicates metabolites not meeting these criteria. Dashed lines denote the thresholds $|\log_2 FC| = 1$ and FDR = 0.05. $\log_2 FC$ was calculated as $\log_2[(\text{group mean} + 1)/(\text{MPR0 mean} + 1)]$ and capped at ±10.
